# Trehalose itself plays a critical role on lipid metabolism: Trehalose increases jejunum cytoplasmic lipid droplets which negatively correlated with mesenteric adipocyte size in both HFD-fed trehalase KO and WT mice

**DOI:** 10.1186/s12986-020-00443-1

**Published:** 2020-03-18

**Authors:** Chikako Arai, Aki Suyama, Shigeyuki Arai, Norie Arai, Chiyo Yoshizane, Satomi Koya-Miyata, Akiko Mizote, Shin Endo, Toshio Ariyasu, Hitoshi Mitsuzumi, Shimpei Ushio

**Affiliations:** Hayashibara Co., Ltd., 675-1 Fujisaki, Naka-ku, Okayama, 702-8006 Japan

**Keywords:** Trehalose, Adipocyte size, Cytoplasmic lipid droplets, Jejunum, Trehalase KO

## Abstract

**Background:**

Trehalose is a functional disaccharide that has anti-metabolic activities such as suppression of adipocyte hypertrophy in mice and alleviation of impaired glucose tolerance in humans. Trehalase hydrolyzes trehalose in the small intestine into two glucose molecules. In this study, we investigated whether trehalose can suppress adipocyte hypertrophy in mice in the presence or absence of trehalase.

**Methods:**

Trehalase knockout (KO) mice and wild-type (WT) mice were fed a high fat diet (HFD) and administered water with 0.3% (w/v) or without trehalose for 8 weeks. At the end of the experimental period, mesenteric adipose tissues and the small intestine were collected and the adipocyte size and proportion of cytoplasmic lipid droplets (CLDs, %) in jejunum epithelium were measured by image analysis.

**Results:**

Trehalose treatment was associated with suppressed adipocyte hypertrophy in both trehalase KO and WT mice. The rate of CLDs in the jejunal epithelium was increased in both trehalase KO and WT mice given water containing trehalose relative to untreated control mice. There was a negative correlation between jejunal epithelial lipid droplet volume and mesenteric adipocyte size. Chylomicron-TG tended to be decreased in both trehalose-treated trehalase KO and WT mice. Addition of trehalose to differentiated Caco-2 cells in vitro increased intracytoplasmic lipid droplets and decreased secretion of the chylomicron marker ApoB-48. Moreover, the jejunal epithelium containing lipid droplets falled into the intestinal lumen, and triglyceride (TG) levels in feces tended to be higher in the KO/HFD/Tre group than in the KO/HFD/Water group. Since then, the accumulation of CLDs has been reported to suppress CM secretion, and along with our results, the effect of trehalose to increase jejunum CLDs may induce adipocyte hypertrophy.

**Conclusions:**

The suppression of adipocyte hypertrophy in the presence and absence of trehalase indicates that trehalose mediates effects prior to being hydrolyzed into glucose. In both trehalase KO and WT mice, trehalose treatment increased the rate of CLDs in jejunal epithelium, reduced chylomicron migration from the intestinal epithelium to the periphery, and suppressed adipocyte hypertrophy. Thus, trehalose ingestion could prevent metabolic syndrome by trapping fat droplets in the intestinal epithelium and suppressing rapid increases in chylomicrons.

## Background

We have continuously been reported that trehalose suppresses adipocyte hypertrophy and mitigates insulin resistance [1–3]. We also demonstrated that trehalose induces white adipose tissue (WAT) browning with suppressing white adipocyte hypertrophy, increasing body temperature, and reducing blood glucose levels even under normal dietary conditions [4]. Trehalose has an inhibitory effect on adipocyte hypertrophy that is not seen for other saccharides. But in the upper small intestine, trehalose is hydrolyzed into two molecules of glucose by trehalase. Trehalase is an intrinsic glycoprotein in the small intestine and renal membranes of animals [5–8] and is involved in glucose transport across the brush-border membranes in the kidney and hydrolysis of ingested trehalose in the intestine [8]. Oku et al. [9] have reported that Japanese subjects are divided into two groups, with low and high trehalase activity. Mizote et al. [10] reported that ingestion of 10 g /day trehalose improved glucose tolerance in human subjects for 12 weeks. They described the following in the discussion. To examine the degree which trehalase activity affected glucose tolerance in their subjects, they conducted a trehalose administration test on the same subjects (data not shown). The subjects were divided into two groups with lower and higher blood glucose elevation after trehalose administration as reported by Oku et al. As a result, glucose tolerance was similarly improved after administration of trehalose for 12 weeks in both groups. These results suggested that trehalose could mediate its effects prior to being hydrolyzed by trehalase.

Oral administration of 1 g/kg trehalose to mice for 5 consecutive days was previously shown to be associated with a significant decrease in the total number of Peyer’s patch (PP) lymphocytes and suppression of spontaneous release of interleukin-6 (IL-6) [11]. Furthermore, Kikusato et al. [12] reported that juvenile chicks fed a diet supplemented with 0.5% trehalose for 18 days showed reduced expression of intestinal inflammatory genes such as interferon-γ and tumor necrosis factor-like ligand 1A. Thus, we assumed that trehalose had some effect on small intestine and trehalose-mediated suppression of adipocyte hypertrophy might be triggered in the small intestine.

We previously generated trehalase-deficient mice using a gene-targeting procedure [13] and deposited these mice with RIKEN. An oral trehalose tolerance test revealed that these trehalase-deficient mice exhibited no changes in blood glucose levels. These mice are thus useful tools for analyzing the mechanisms of trehalose action. Murotomi et al. [14] reported that 1.5% of trehalose drinking group strongly mitigate glucose tolerance in Tsumura Suzuki Obese Diabetes (TSOD) mice compared with 0.3% trehalose (w/v) group. So, we hypothesized that trehalase KO mice would show a stronger effect of trehalose compared to WT mice. Here we used trehalase KO mice and WT mice to investigate whether trehalose can suppress adipocyte hypertrophy and elicit changes in intestinal function in the presence or absence of trehalase.

## Materials and methods

### Animals

Ten-weeks-old female trehalase KO mice (RBRC00857, background strain C57BL/6 J) and WT mice were obtained from the RIKEN BioResource Research Center (Tsukuba, Japan) and fed a standard diet (CE-2; CLEA Japan, Inc.) and water ad libitum for 2 weeks. The mice were kept in a temperature-controlled room with a 12-h light cycle. This study was approved by the Laboratory Animal Care Committee of the Hayashibara Co., Ltd. (Okayama, Japan) and all experiments involving animals were conducted in accordance with the Guidelines for Care and Use of Laboratory Animals of the Hayashibara Co., Ltd.

### Test substance

Trehalose (Reagent grade; Hayashibara Co., Ltd.) containing > 98.0% trehalose dihydrate was used as the source of trehalose.

### Study design

**Fig. 1:**
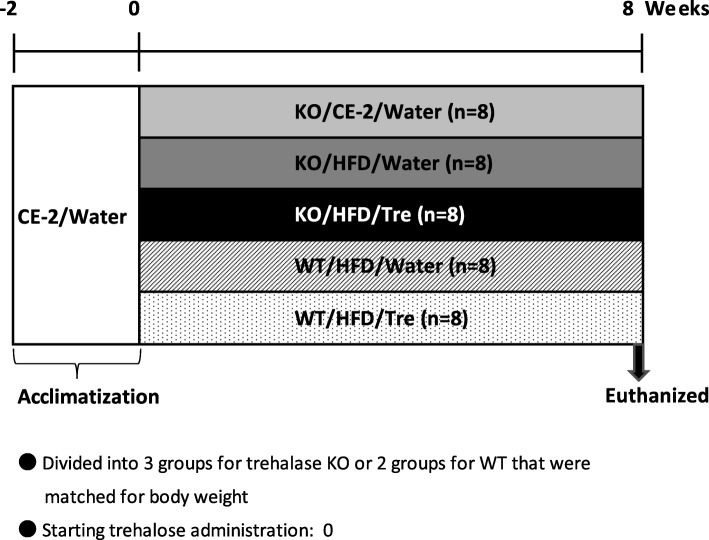
Experimental protocol. After 2 weeks of acclimatization, trehalase KO mice were divided into 3 groups and WT mice were divided into 2 groups matched by body weight and then administered with 0.3% (w/v) trehalose in the drinking water or water alone. The mice were euthanized after 8 weeks. Histological examination of the intestine was carried out and measurement of adipocyte size and the rate of jejunal lipid accumulation was conducted by image analysis and serum lipid analysis, respectively

The experimental protocol is shown in Fig. 1. A total of 40 mice were acclimated for 2 weeks and then the 12-weeks-old mice were randomly divided into 5 groups and matched for average body weight. Two groups of trehalase KO mice and two groups of WT mice were fed a high fat diet (HFD32, CREA Co., Ltd., Japan) and then given drinking water *ad libitum *that either lacked (control) or contained 0.3% (w/v) trehalose (KO/HFD/Water, KO/HFD/Tre, WT/HFD/Water, WT/HFD/Tre, *n* = 8 for each group). As the experimental control between trehalase KO group, another group were given a normal diet and water *ad libitum *(KO/CE-2/Water, n = 8). Four mice were grouped per cage with each cage containing animals from a single experimental group. Food and water were replaced every other day and food intake was monitored; body weights were recorded weekly throughout the experimental period. After 8 weeks of treatment, the animals were euthanized under pentobarbital anesthesia. The adipose tissue was weighed, and blood samples were collected from the abdominal vena cava for measurement of serum lipids and chylomicrons (CM). Serum TG and non-esterified fatty acids (NEFA) were measured using Triglyceride E-test kits and NEFA C-test kits (FUJIFILM Wako Pure Chemical Corporation, Osaka, Japan), respectively. CM was measured by high sensitivity gel filtration HPLC (Skylight-biotech, Akita, Japan).

### Calculation of energy intake

The energy densities of the HFD, a normal diet, and the drinking water containing 0.3% (w/v) trehalose were 20.9 kJ, 14.2 kJ, and 0.05 kJ per gram, respectively. Based on these data, the mean energy intake per mouse in each group was calculated using the following formulas:

Energy intake (kJ/mouse/day):

Mice consuming HFD and 0.3% (w/v) trehalose:

Food intake (g) × 20.9 (kJ) + water intake (g) × 0.05 (kJ).

Mice consuming HFD and water:

Food intake (g) × 20.9 (kJ) + water intake (g) × 0 (kJ).

Mice consuming normal diet and water:

Food intake (g) × 14.2 (kJ) + water intake (g) × 0 (kJ).

### Histological analysis of adipocyte size

Mesenteric adipose tissue samples were fixed in 10% (v/v) buffered formalin and embedded in paraffin. The sections were deparaffinized with xylene, stained with hematoxylin and eosin, and then examined by light microscopy. Photographs of 5 random areas per section in the respective adipose tissue were taken at 200 × magnification. More than 200 adipocyte sizes were measured by image analysis software (cellSens, Olympus Corporation, Tokyo, Japan).

### Rates of CLDs in jejunal epithelium

Sections of intestine were stained with hematoxylin and eosin, and then examined using light microscopy. Jejunal cytoplasmic vacuoles were demonstrated to contain neutral lipids by Oil Red O staining of frozen sections. Photographs of 5 random areas in the respective jejunal section were taken at 400x magnification. The proportion of area containing lipid (%) was measured in more than 20 intestinal villi/mouse by image analysis software (cellSens).

### Cell culture

We prepared Caco-2 cells, a human colon carcinoma cell line, according to the method described by Vidal et al. [15] and Morel et al. [16]. The cells were seeded at 1.5 × 10^4^cells/well in a 24-well insert cup (0.4 μm pore size, polyethylene terephthalate) treated with atelocollagen, and cultured for 1 week in Dulbecco’s modified Eagle’s medium (DMEM) containing 20% fetal bovine serum (FCS) at 37 °C. Confluency was confirmed by measuring electric resistance before serum-free DMEM and DMEM containing 20% FCS DMEM were added to the apical and basal side, respectively. The cells were cultured for an additional week to promote differentiation into intestinal epithelial cells, which were then divided into groups according to the electrical resistance values before use in experiments.

### Micelle treatment of Caco-2 cells

As reported by Hernell et al. [17], micelles in the duodenum consist of 0.3 mM oleic acid, 0.025 mM cholesterol, 0.1 mM 2-monooleylglycerol, 1.0 mM taurocholic acid and 0.1 mM α-lysophosphatidylcholine. Lipids at these concentrations were dissolved in ethanol in a glass test tube, dried with N_2_ gas, and stored at − 80 °C until use. After adding serum-free DMEM medium to the lipids and sonicating for 20 min, they were mixed with the same amount of serum-free DMEM medium with or without trehalose (50 mM final concentration) and sonicated for 5 min. The medium on the apical side of the differentiated Caco-2 cells was removed, and 300 μL/well of the micelle solution was added. After incubating for 24 h, basal side culture medium was collected in a tube, mixed with polyoxyethylene (10) octylphenyl ether and EDTA at a final concentration of 1% and 5 mM, respectively, and protein inhibitors before storage at − 80 °C until use. When assessing the amount of intracellular lipid accumulation, the content of micelle components was reduced by half to prevent cytotoxicity and mixed with the fluorescently labeled fatty acid BODIPY™ FL C_16_ (Thermo Fisher Scientific Inc., Waltham, MA, USA) at a final concentration of 10 μM.

### Measurement of lipid droplet accumulation in Caco-2 cells

The cultured cells were fixed by treatment with 4% formaldehyde for 5 min. The membrane at the bottom of the insert cup was cut with a scalpel, transferred to a slide glass, and encapsulated with mounting medium (50% glycerol, 0.05% NaN_3_ in phosphate buffered saline). Then, the specimen was observed with a fluorescence microscope (Olympus BX53-FK, exposure time: 10 milliseconds), and the ratio of BODIPY™ FL C_16_ that accumulated in areas containing lipid droplets was analyzed with Olympus cellSens.

### Evaluation of secreted ApoB-48 and ApoB-100 by western blotting

Basal side culture medium was collected and mixed with × 1.25 sample buffer (2.08% sodium lauryl sulfate, 6.25% glycerol, 1.94% dithiothreitol in 0.073 M Tris-HCl buffer, pH 6.8) and denatured by boiling for 5 min. The protein was subjected to 5% SDS-PAGE and transferred to a PVDF membrane. The membrane was treated with blocking agent, and labeled with a mouse anti-human ApoB monoclonal antibody (7B8) (ab39560, Abcam plc, Cambridge, UK) as the primary antibody, and horseradish peroxidase-labeled goat anti-mouse IgG polyclonal antibody (Nr.P.0447, Dako, Agilent Technologies, Santa Clara, CA) as the secondary antibody, which was detected by ECL prime detection agent (GE Healthcare, Little Chalfont, UK). The resulting images were analyzed with Image J (version 1.52a).

### Statistics

Data are expressed as means ± standard deviations. A power analysis (G*Power 3.1.9.4, http://www.gpower.hhu.de/) showed that a sample size of 8 mice per group was suitable to detect a difference between 5 experimental groups (1-β = 0.95, effect size = 0.8, α = 0.01). In addition, the calculated *p* values were described. Statistically significant effects of trehalose on dispersion uniformity and normality were examined using Tukey-Kramer (Statcel, ver. 3). Non-parametric data were analyzed by the Steel-Dwass test (Statcel, ver. 3). A *p*-value less than 0.05 was considered significant.

## Results

### Continuous ingestion of trehalose had no effect on energy intake, body weight, tissue weight, and serum lipid

The energy intake values for trehalase KO and WT mice given water with or without trehalose during the experimental period were similar at 59.8 ± 4.7, 59.0 ± 4.2, 58.2 ± 4.5, 55.2 ± 5.1, and 56.0 ± 3.0 kJ per mouse per day in the KO/CE-2/Water, KO/HFD/Water, KO/HFD/Tre, WT/HFD/Water, and WT/HFD/Tre groups, respectively. Furthermore, the four HFD groups exhibited no significant differences in overall body weight, or weight of adipose tissue, serum NEFA. Body weight and fat weight were lower in WT/HFD/Water than those in KO/HFD/water, but not significant. On the other hand, liver weight was significantly lower in the WT/HFD/Water group compared to the KO/HFD/Water group (Table 1).

**Table 1:**
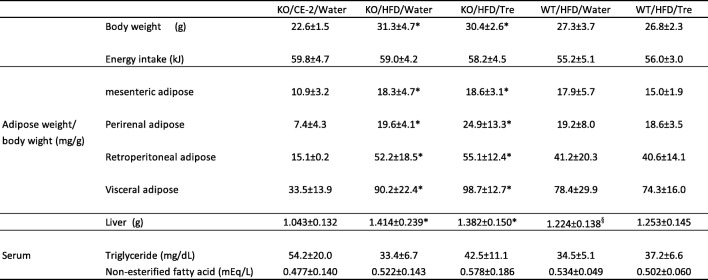
Energy intake, body, organ weights, serum lipids in mice after 8 weeks of trehalose intake.

Values are shown as means ± SD for 7–8 mice per group. There were 8 mice in the KO/CE-2/Water and WT/HFD/Water groups and 7 mice in the other groups. Statistical analysis was performed using a Steel-Dwass test. *Statistically significant (*p* < 0.05) difference compared to the KO/CE-2/Water group; ^§^Statistically significant (*p <* 0.05) difference between the KO/HFD/Water and WT/HFD/Water group

### Trehalose suppressed adipocyte hypertrophy in mesenteric adipose tissues

**Fig. 2:**
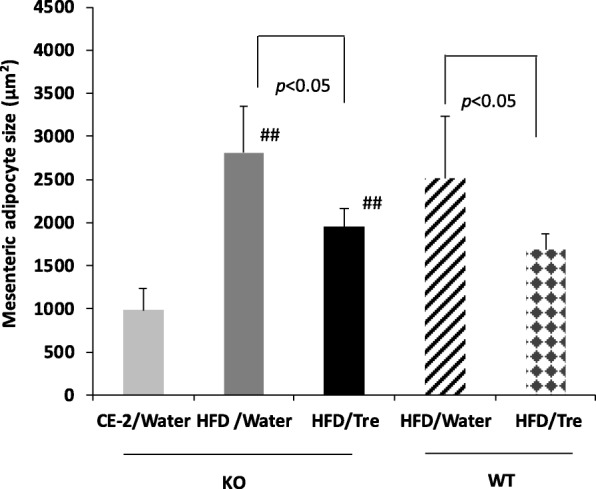
Effect of drinking water containing trehalose on the size of mesenteric adipocytes. The adipocyte size was measured using cellSens image software. Values are shown as means ± standard deviation (*n* = 7–8). Statistical analysis was performed with a Tukey-Kramer test. The values indicate that the statistical significances were *p* < 0.05 and *p* < 0.01, respectively. KO/HFD groups vs. KO/CE-2/Water: ##*p* < 0.01

We examined the histology of the mesenteric adipose tissues and measured the adipocyte sizes from both trehalase KO and WT mice groups (with and without trehalose in the water) using cellSens imaging software. For trehalase KO mice, the size of mesenteric adipocytes from the HFD/Tre group (1953 ± 209 μm^2^) was significantly smaller than that for the HFD/Water group (2809 ± 541 μm^2^; *p* < 0.05; Fig. 2). The WT/HFD/Tre group (1683 ± 189 μm^2^) had significantly smaller mesenteric adipocytes than for the WT/HFD/Water group (2515 ± 717 μm^2^, *p* < 0.05). Between the KO and WT mice groups with the same treatment, WT mice tended to have smaller adipocytes, but were not significant. Moreover, the effect of trehalose on adipocyte hypertrophy was similar between the trehalase KO and WT mice.

### Trehalose increased the proportion of CLDs in jejunum epithelium

**Fig. 3:**
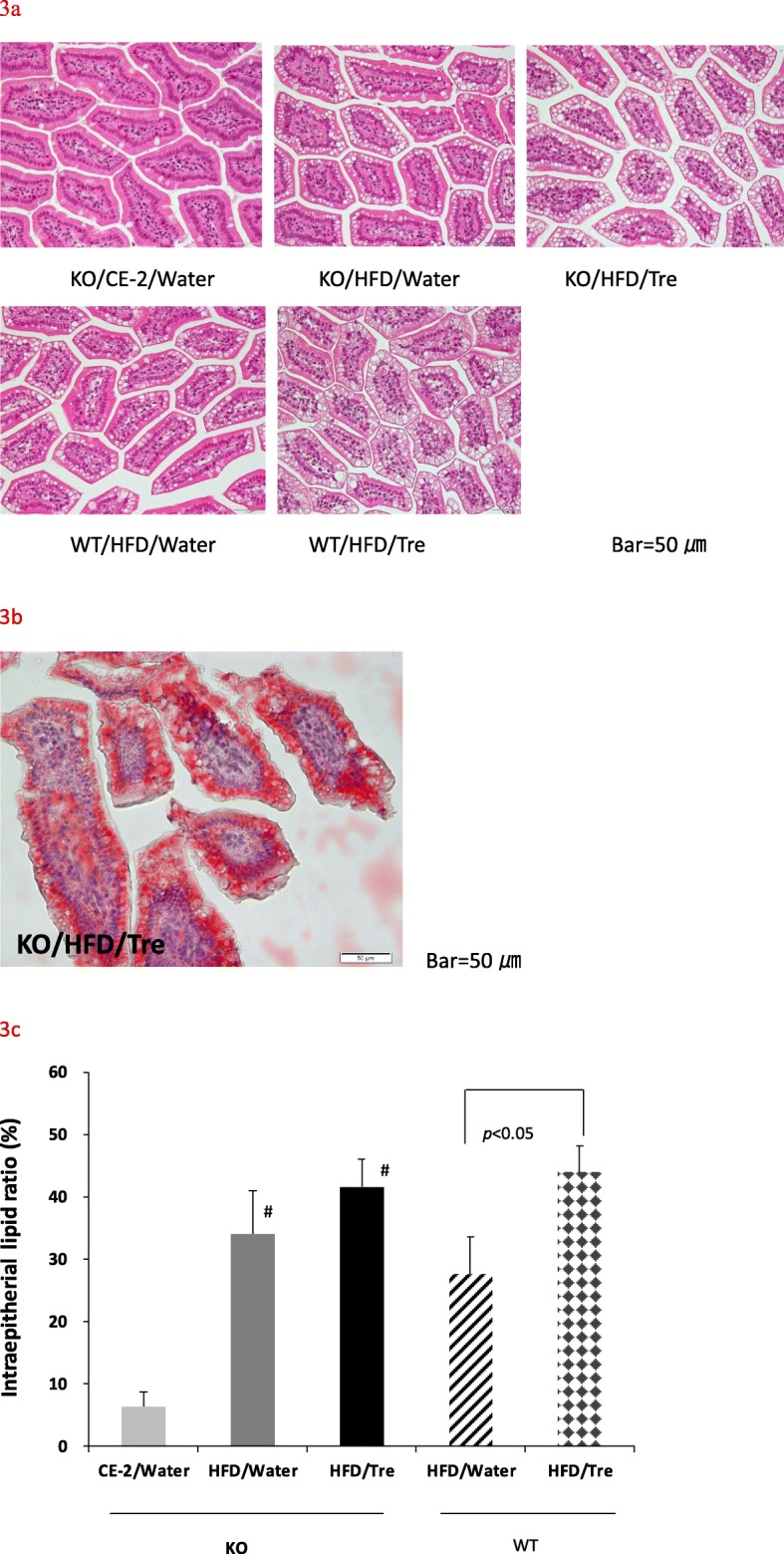
Effect of drinking water containing trehalose on intracytoplasmic lipid droplets in jejunal epithelium. Representative images (400x) of hematoxylin-eosin staining in sections of jejunum are shown (**a**) Oil Red O staining showing the presence of neutral lipids in jejunal intracytoplasmic vacuoles (**b**) intraepithelial lipid ratio (%) in the jejunal epithelium determined using the image software cellSens (**c**). Values are shown as means ± standard deviation (*n* = 7–8). Statistical analysis was performed using a Steel-Dwass test. The values indicate that the statistical significances were *p* < 0.05. KO/HFD groups vs. KO/CE-2/Water: #*p* < 0.05

Histopathological examination of the entire intestine of the trehalase KO and WT mice showed that intracytoplasmic vacuole was most intense in the upper jejunum. In both the trehalase KO and WT mice, the number of intracytoplasmic vacuoles were markedly increased in the groups given trehalose (Fig. 3a). Vacuoles of the jejunal epithelium were demonstrated to contain neutral lipids by Oil Red O staining of frozen sections (Fig. 3b). Then, we determined intracytoplasmic vacuoles as CLDs.

**Fig. 4:**
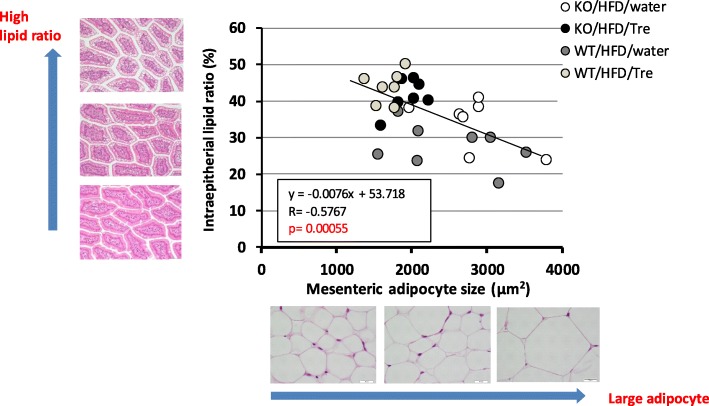
Correlation between intraepithelial lipid ratio (%) and mesenteric adipocyte size. There was a negative correlation between mesenteric adipocyte size and jejunal intraepithelial lipid ratio (%) (*n* = 7–8, each group: Pearson correlation coefficients r = − 0.57). The value indicates statistical significance (*p* < 0.01)

We also measured the proportion of CLDs in the jejunum by image analysis. Trehalase KO mice given water containing trehalose tended to have an increased proportion of CLDs (41.6 ± 4.5%) compared to the water only group (34.1 ± 6.9%). Moreover, in WT mice, the proportion of CLDs in the trehalose group (44.0 ± 4.3%) was significantly higher than that of the water group (27.7 ± 5.9%) (Fig. 3c). The ratio of intraepithelial lipid was slightly higher in the KO/HFD/water group than in the WT/HFD/Water group, but the ratio was almost the same by trehalose drinking. Surprisingly, a negative correlation was observed between the proportion of CLDs (%) in the jejunum and the size of mesenteric adipocytes across the HFD groups (R = -0.57, *p* < 0.01) (Fig. 4). Whereas the adipocytes in the water only group exhibited various sizes in both trehalase KO and WT mice, in the groups given water containing trehalose, most of animals had small adipocyte size and a high proportion of CLDs. Mice with small adipocytes had many CLDs in the jejunum.

### Trehalose promoted detachment of lipid droplets from the intestinal epithelium to the intestinal lumen and the lipids were excreted in the feces

**Fig. 5:**
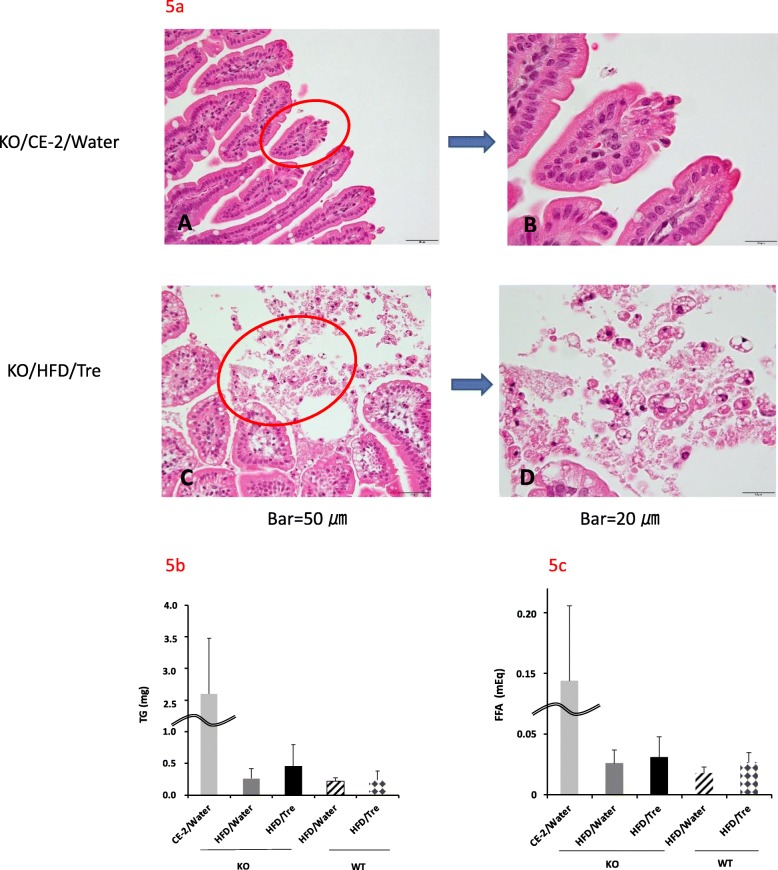
Effect of drinking water containing trehalose on fecal lipid excretion. Detached jejunal microvillus to the lumen of KO/CE-2/Water (**a-A**), and KO/HFD/Tre (**a**-**C**) and magnified images of jejunal microvillus (**a-B**, −**D**) are shown. The amount of TG and FFA excreted in feces are summarized in 5b and 5c, respectively. Values are shown as means±standard deviation (*n* = 7–8). Statistical analysis was performed using Steel-Dwass test. No statistical significance was observed between water group and Tre group, in both trehalase KO and WT mice

Intestinal epithelium is reported to turn over every 3–4 days [18]. When we observed jejunum microvilli, the intestinal epithelium contained many lipid droplets that were detached in the intestinal lumen of mice fed HFD compared to mice fed a normal diet (Fig. 5a). We also measured TG and FFA excreted in feces. In the trehalase KO mice, the amount of TG excreted in the feces of the trehalose group was slightly increased compared to that for the water group (Fig. 5b). KO/HFD/Tre tended to have a higher TG than WT/HFD/Tre. In both the trehalase KO and the WT mice, FFA excreted in feces of the trehalose groups tended to be increased relative to that for the water groups (Fig. 5c). Since the daily fecal weight for the groups fed a normal diet was 7-fold higher than that for the HFD group, the amount of lipid excretion in the normal group was apparently increased.

### Trehalose decreased serum CM-TG

**Fig. 6:**
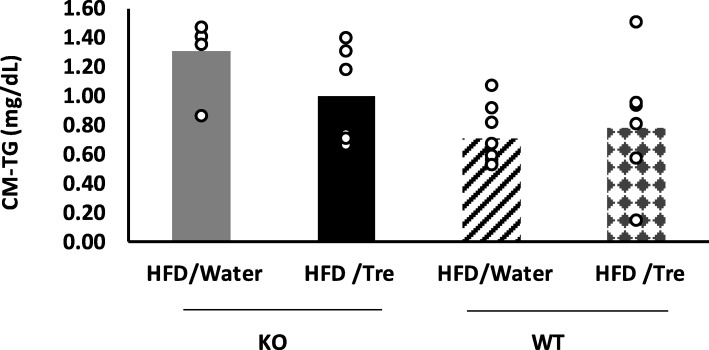
Effect of drinking water containing trehalose on serum CM-TG concentration. Serum CM-TG was measured by high sensitivity gel filtration HPLC. The individual value was plotted (*n* = 5–8). No statistical significance was observed

We next measured serum CM-TG levels to examine whether the amount of CM-TG was decreased by jejunal lipid droplet trapping. In both the trehalase KO and WT mice, the amount of serum CM-TG tended to decrease in the trehalose group compared to the water group. CM-TG was lower in WT/HFD/Water than in KO/HFD/Water. (Fig. 6).

### Inhibitory effect of ApoB48 secretion by trehalose in vitro

**Fig. 7:**
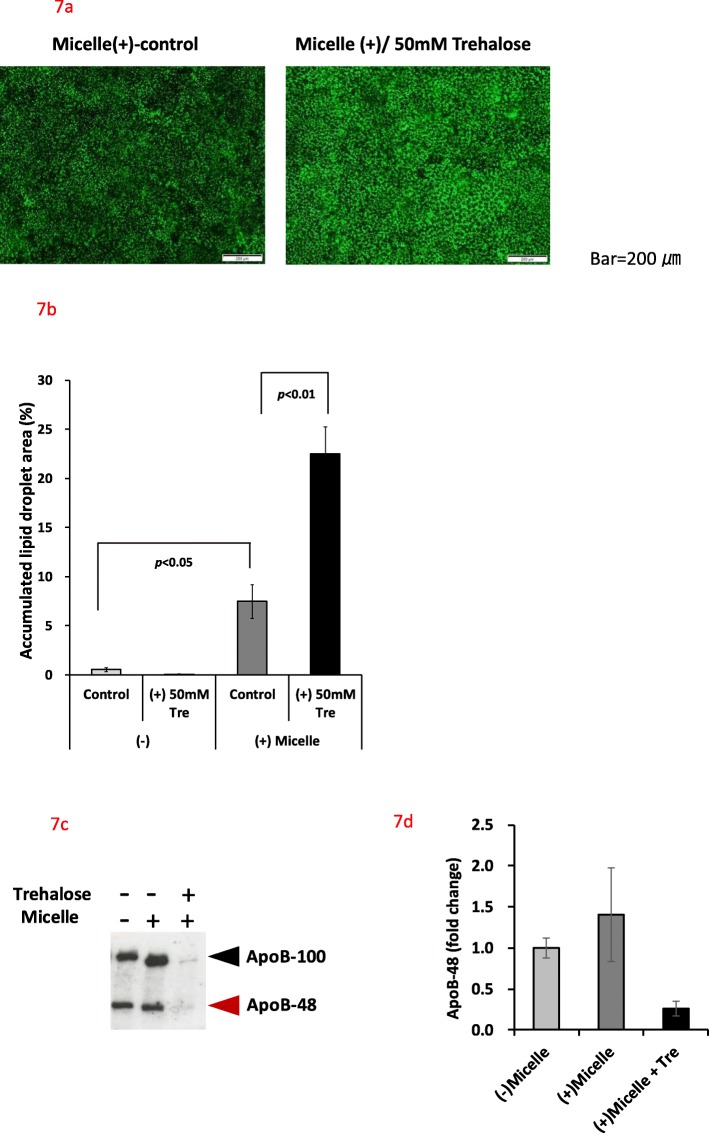
Effect of trehalose on lipid droplet accumulation, ApoB-100 and ApoB-48 secretion in Caco-2 cells. Caco-2 cells were treated with or without micelles and 50 mM trehalose. The cultured cells were fixed with formaldehyde, mounted and observed using a fluorescence microscope (**a**). The bright areas with BODIPY™ FL C_16_ were analyzed (**b**). Culture supernatants from the basal side were analyzed for ApoB-100 and ApoB-48 by western blotting (**c**). The relative amount of ApoB-48 with or without Tre relative to micelles (−) was summarized (**d**). Values are shown as means ± standard deviation (*n* = 3). The values indicate statistical significance (7b: *p* < 0.05, *p* < 0.01, Tukey-Kramer test). No statistical significance was observed (7d: t-test)

Caco-2 cells differentiated into intestinal epithelium cells were treated with lipid micelles for 24 h, and the lipid droplet area accumulated in the cells was measured (Fig. 7a). In the absence of micelles, the lipid droplet area in the control group (0.52 ± 0.21%) and 50 mM Tre-treated group (0.05 ± 0.02%) was similar. Upon micelle treatment, the area increased to 7.46 ± 1.72%. When cells were treated with both micelles and 50 mM Tre, the lipid droplet area increased to 22.51 ± 2.74%, approximately three times that of the control group treated with micelles only (Fig. 7b). The differentiated Caco-2 cells were subsequently treated with the micelles for 24 h, and the amount of ApoB-48 secreted in the basal culture medium was measured (Fig. 7c, d). The micelle treatment increased the amount of ApoB-48 secretion on the basal side, and this secretion was markedly suppressed in the presence of 50 mM Tre.

## Discussion

In the present study, we demonstrated that trehalose suppressed adipocyte hypertrophy and there was a negative correlation between the size of mesenteric adipocytes and the proportion of jejunal CLDs in both WT and trehalase KO mice. To our knowledge, this is the first study to assess the effect of HFD on adipocyte hypertrophy and accumulation of jejunal CLDs in trehalase KO mice. Comparing the water group of trehalase KO and WT mice with or without trehalase, phenotypes such as body weight, adipocyte hypertrophy, and the proportion of jejunal CLDs did not change.

D’Aquila et al. [19] reviewed the significance of jejunal CLDs. Dietary fat consumed as TG is efficiently digested into fatty acids (FA) in the gastrointestinal lumen and absorbed by enterocytes. The digested products taken up by enterocytes are re-synthesized into TGs and packaged either in chylomicrons (CMs) for secretion or in CLDs for storage. Although the CLDs were thought to be an inactive reservoir of neutral lipids, they are now recognized as dynamic organelles that have functions beyond lipid metabolism [20, 21]. The synthesis of CLDs is thought to buffer enterocytes from FA toxicity and regulate the rate of synthesis and secretion of CMs [19]. While all regions of the small intestines can take up and absorb digestive products of TG, the jejunum is the major site for TG metabolite uptake and absorption [22, 23].

When we examined whole intestine tissues histopathologically, the upper jejunum had the strongest intensity of CLD staining and the result was consistent with previous reports [22, 23]. The finding of substantially more jejunal lipid droplets in the trehalose group compared to the water group was unexpected. Since the accumulation of CLDs suppresses the secretion of CM that migrates to the lymphatic system, according to the review by D’Aquila et al. [19], intestinal CLDs would not necessarily be detrimental. Moreover, this review also reported that the amount of CLDs increased after meals and decreased by fasting due to enterocyte turnover, and that malabsorption of fat in stool did not occur under normal physiological conditions. We also conducted histopathological evaluation of the intestine, liver and pancreas, but we observed no abnormal changes associated with trehalose treatment. In particular, there were no differences between the HFD groups in the number of intestinal villi and villi length in specific areas. In contrast, there was a negative correlation between the size of mesenteric adipocytes and the proportion of jejunal CLD content. Trapping lipid droplets in the upper jejunum could be involved in the induction of adipocyte hypertrophy suppression.

Soriguer et al. [24] reported that plasma levels of glucose, insulin, TG, CM, ApoB-48, Apo A-IV levels and HOMA-IR were all significantly higher in morbidly obese patients with T2DM. But the jejunal wall TG concentration in these patients was markedly lower than in morbidly obese patients without T2DM. In this study, amounts of ApoB-48, CM-TG, and HOMA-IR were negatively correlated with jejunal TG. Therefore, the difference between diabetes and obesity may be due to the suppression of a transition from jejunal lipid droplet traps to CM. These findings were consistent with data of our study.

Trehalose increased jejunum CLDs and it induced decrease of CM secretion into bloods and suppressed adipocyte hypertrophy. The amount of ApoB-48 that passed under the basement membrane of Caco-2 cells were measured for the inhibitory effects of trehalose on CM secretion. A decrease in the amount of ApoB-48 was observed in the presence of trehalose. Since ApoB-48 is a component protein of CM, CM secretion could be suppressed by trehalose. The results were consistent with the mouse experiment.

Although there was a tendency to suppress CM secretion in animal tests, unfortunately, there was no significant difference. In addition, it was thought that CLDs trapped in the intestine was excreted in feces by intestinal epithelial detachment, TG and FFA in feces tended to increase with trehalose, but not significant. Furthermore, trehalose increases the lipids excretion into feces from CLDs, but it is not enough to explain the consequences of CLDs, so it is necessary to examine whether lipolysis or autophagy is enhanced or not.

Comparison of trehalase KO and WT mice fed HFD indicated that trehalose had nearly similar effects on suppression of adipocyte hypertrophy and on the jejunal CLD trap rate. Since trehalose is hydrolyzed by trehalase in the upper small intestine, the amount of trehalose that reaches the lower small intestine is higher in the trehalase KO mice than the WT mice. However, the effects of trehalose on adipocyte size and jejunal lipid droplet trapping were nonetheless equivalent. The similarity in the effects of trehalose on WT and trehalase KO mice suggested that the trehalose-mediated action occurred before trehalose was hydrolyzed by trehalase.

Xiao et al. [25] performed a single lipid challenge test in which glucose or water was consumed 5 h after a high fat liquid meal challenge, and a duodenal biopsy was performed 1 h later to compare the amount of CLDs. Their data indicate that oral glucose mobilizes TGs stored within enterocyte CLDs and provides a substrate for CM synthesis and secretion. At 1 h after glucose ingestion, the amount of CM-TG was significantly higher and the number of CLDs in the jejunum was significantly lower compared to the water group. This result suggested that glucose suppressed CLD accumulation in the intestine and they were instead rapidly transferred to lymphatic vessels as CM. When glucose was given to mice fed a HFD in our study, we found no suppression of adipocyte hypertrophy [1]. This phenomenon was apparently different from the effect of trehalose on CLDs. Trehalose and glucose did the opposite action, despite glucose being a hydrolyzed product of trehalose. Therefore, the trehalose-mediated action occurs before trehalose is degraded by trehalase and thus these effects could be induced in sites such as the oral cavity, stomach, duodenum and upper jejunum.

We thought about why trehalose increases intestinal CLDs. Auclair et al. [26] reviewed several mechanisms are proposed to influence CLD formation in the intestine, a plethora diet (e.g. high fat/sucrose diet), intracellular lipid transporters (e.g. CD36), GIP peptide (e.g. GLP-2), intracellular signaling systems (e.g. Clock genes, Hedgehog), transcription factors (e.g. liver-x receptor a: LXRa) and gut microbiota are proposed to modulate the formation and hydrolysis of CLDs in the intestine.

Moreover, Auclair et al. [26] reported that increased intestinal AMPK activity regulates lipolysis and decreased secretion of CM from CLDs. It was reported that HMW-adiponectin increase AMPK activity in liver and muscles [27] and 3 T3-L1 adipocyte [28]. In our previous study, trehalose maintained high serum HMW-adiponectin levels in HFD-fed mice compared to the water group [2]. This result suggests that trehalose maintains high levels of HMW-adiponectin that in turn activates AMPK. Then, activated intestinal AMPK activation may increase CLDs. We will need to investigate whether there are differences in intestinal AMPK activity with trehalose treatment of mice in future studies.

## Conclusions

Contrary to the hypothesis, our study first showed that trehalose itself plays an important role on lipid metabolism, as compared with trehalase KO and WT mice. When trehalose was ingested in mice loaded with the HFD, we observed that lipid droplets were trapped in the jejunum epithelium and then the intestinal epithelium was exfoliated, lipids were excreted in feces, and the amount of fat transferred to peripheral adipose tissue as chylomicron decreased. These results, together with a negative correlation between jejunal epithelial lipid droplet volume and mesenteric adipocyte size, suggest that these phenomena would suppress adipocyte hypertrophy. Furthermore, our results suggest that prevention of rapid migration of lipids into the blood and peripheral tissues may have a preventive effect on metabolic syndrome.

## Data Availability

All data generated and analyzed during this study are included in the manuscript.

## Abbreviations

AMPK: AMP-activated protein kinase
ApoB48: Apolipoprotein B48
CLDs: Cytoplasmic lipid droplets
CM: Chylomicron
CM-TG: Chylomicron-triglyceride
FFA: Free fatty acids
HFD: High fat diet
HMW-adiponectin: High molecular weight-adiponectin
IL-6: Interleukin-6
KO: Knockout
KO/CE-2/Water: Trehalase KO mice fed normal diet with water
KO/HFD/Tre: Trehalase KO mice fed high fat diet with trehalose
KO/HFD/Water: Trehalase KO mice fed high fat diet with water
LXRa: Liver-x receptor a
PP: Peyer’s patch
TG: Triglyceride
TSOD: Tsumura Suzuki Obese Diabetes
WAT: White adipose tissue
WT/HFD/Tre: Wild type mice fed high fat diet with trehalose
WT/HFD/Water: Wild type mice fed high fat diet with water
w/v: Weight/volume
v/v: Volume/volume

## References

[1] Arai C, Arai N, Mizote A, Kohno K, Iwaki K, Hanaya T, Arai S, Ushio S, Fukuda S (2010). Trehalose prevents adipocyte hypertrophy and mitigates insulin resistance. Nutr Res.

[2] Arai C, Miyake M, Matsumoto Y, Mizote A, Yoshizane C, Hanaya Y, Koide K, Yamada M, Hanaya T, Arai S, Fukuda S (2013). Trehalose prevents adipocyte hypertrophy and mitigates insulin resistance in mice with established obesity. J Nutr Sci Vitaminol (Tokyo).

[3] Arai C, Miyata M, Yoshizane C, Koide K, Mizote A, Arai N, Hanaya T, Arai S, Fukuda S (2013). Trehalose protect islets of Langerhans in HFD-fed obese mice: a morphometric analysis. J Jpn Soc Nutr Food Sci.

[4] Arai C, Arai N, Arai S, Yoshizane C, Miyata S, Mizote A, Suyama A, Endo S, Ariyasu T, Mitsuzumi H, Ushio S (2019). Continuous intake of trehalose induces white adipose tissue browning and enhances energy metabolism. Nutr Metabo.

[5] Ishihara R, Taketani S, Sasai-Takedatsu M, Kino M, Tokunaga R, Kobayashi Y (1997). Molecular cloning, sequencing and expression of cDNA encoding human trehalase. Gene..

[6] Oesterreicher TJ, Markesich DC, Henning SJ (2001). Cloning, characterization and mapping of mouse trehalase (Treh) gene. Gene..

[7] Oesterreicher TJ, Nanthakumar NN, Winston JH, Henning SJ (1998). Rat trehalase: cDNA cloning and mRNA expression in adult rat tissues and during intestinal ontogeny. Am J Phys.

[8] Sacktor B (1968). Trehalase and the transport of glucose in the mammalian kidney and intestine. Proc Natl Acad Sci U S A.

[9] Oku T, Nakamura S (2000). Estimation of intestinal trehalase activity from laxative threshold of trehalose and lactulose on healthy female subjects. Eur J Clin Nutr.

[10] Mizote A, Yamada M, Yoshizane C, Arai N, Maruta K, Arai S, Endo S, Ogawa R, Mitsuzumi H, Ariyasu T, Fukuda S (2016). Daily intake of trehalose is effective in the prevention of lifestyle-related diseases in individuals with risk factors for metabolic syndrome. J Nutr Sci Vitaminol (Tokyo).

[11] Arai N, Yoshizane C, Arai C, Hanaya T, Arai S, Ikeda M, Kurimoto M (2002). Trehalose ingestion modifies mucosal immune responses of the small intestine in mice. J Health Sci.

[12] Kikusato M, Nanto F, Mukai K, Toyomizu M (2016). Effects of trehalose supplementation on the growth performance and intestinal innate immunity of juvenile chicks. Br Poult Sci.

[13] Kamiya T, Hirata K, Matsumoto S, Arai C, Yoshizane C, Kyono F, Ariyasu T, Hanaya T, Arai S, Okura T, Yamamoto K, Ikeda M, Kurimoto M (2003). Targeted disruption of the trehalase gene: determination of the digestion and absorption of trehalose in trehalase- deficient mice. Nutr Res.

[14] Murotomi K, Arai S, Suyama A, Harashima A, Nakajima Y (2019). Trehalose attenuates development of nonalcoholic steatohepatitis associated with type 2 diabetes in TSOD mouse. J functional foods.

[15] Vidal R, Hernandez-Vallejo S, Pauquai T, Texier O, Rousset M, Chambaz J, Demignot S, Lacorte JM (2005). Apple procyanidins decrease cholesterol esterification and lipoprotein secretion in Caco-2/TC7 enterocytes. J Lipid Res.

[16] Morel E, Demignot S, Chateau D, Chambaz J, Rousset M, Deler F (2004). Lipid-dependent bidirectional traffic of apolipoprotein B in polarized enterocytes. Mol Biol Cell.

[17] Hernell O, Staggers JE, Carey MC (1990). Physical-chemical behavior of dietary and biliary lipids during intestinal digestion and absorption. 2. Phase analysis and aggregation states of luminal lipids during duodenal fat digestion in healthy adult human beings. Biochemistry..

[18] Park Jung-ha, Kotani Takenori, Konno Tasuku, Setiawan Jajar, Kitamura Yasuaki, Imada Shinya, Usui Yutaro, Hatano Naoya, Shinohara Masakazu, Saito Yasuyuki, Murata Yoji, Matozaki Takashi (2016). Promotion of Intestinal Epithelial Cell Turnover by Commensal Bacteria: Role of Short-Chain Fatty Acids. PLOS ONE.

[19] D’Aquila T, Hung YH, Carreiro A, Buhman KK (1861). Recent discoveries on absorption of dietary far: presence, synthesis, and metabolism of cytoplasmic lipid droplets within enterocytes. Biochim Biophys Acta.

[20] Gross DA, Silver DL (2014). Cytosolic lipid droplets: from mechanisms of fat storage to disease. Crit Rev Biochem Mol Biol.

[21] Krahmer N, Farese RV, Walther TC (2013). Balancing the fat: lipid droplets and human disease. EMBO Mol Med.

[22] Zhu J, Lee B, Buhman KK, Cheng JX (2009). A dynamic, cytoplasmic triacylglycerol pool in enterocytes revealed by ex vivo and in vivo coherent anti-stokes Raman scattering imaging. J Lipid Res.

[23] de Wit N, Derrien M, Bosch-Vermeulen H, Oosterink E, Keshtkar S, Duval C, de Vogel-van den Bosch J, Kleerebezem M, Müller M, van der Meer R (2012). Saturated fat stimulates obesity and hepatic steatosis and affects gut microbiota composition by an enhanced overflow of dietary fat to the distal intestine. Am J Physiol Gastrointest Liver Physiol.

[24] Soriguer F, García-Serrano S, Garrido-Sánchez L, Gutierrez-Repiso C, Rojo-Martínez G, Garcia-Escobar E, García-Arnés J, Gallego-Perales JL, Delgado V, García-Fuentes E (2010). Jejunal wall triglyceride concentration of morbidly obese persons is lower in those with type 2 diabetes mellitus. J Lipid Res.

[25] Xiao C, Stahel P, Carreiro AL, Hung YH, Dash S, Bookman I, Buhman KK, Lewis GF (2019). Oral glucose mobilizes triglyceride stores from the human intestine. Cell Mol Gastroenterol Hepatol.

[26] Auclair N, Melbouci L, St-Pierre D, Levy E (2018). Gastrointestinal factors regulating lipid droplet formation in the intestine. Exp Cell Res.

[27] Yamauchi T, Kamon J, Minokoshi Y, Ito Y, Waki H, Uchida S, Yamashita S, Noda M, Kita S, Ueki K, Eto K, Akanuma Y, Froguel P, Foufelle F, Ferre P, Carling D, Kimura S, Nagai R, Kahn BB, Kadowaki T (2002). Adiponectin stimulates glucose utilization and fatty-acid oxidation by activating AMP-activated protein kinase. Nat Med.

[28] Li Y, Wang P, Zhuang Y, Lin H, Li Y, Liu L, Meng Q, Cui T, Liu J, Li Z (2011). Activation of AMPK by berberine promotes adiponectin multimerization in 3T3-L1 adipocytes. FEBS Lett.

